# Mechanisms of inflammation after ischemic stroke in brain-peripheral crosstalk

**DOI:** 10.3389/fnmol.2024.1400808

**Published:** 2024-06-12

**Authors:** Ling Xie, Ming He, Caidi Ying, Haifeng Chu

**Affiliations:** ^1^Department of Critical Medicine, First People’s Hospital of Linping District, Hangzhou, China; ^2^Department of Hepatobiliary and Pancreatic Surgery, The Traditional Chinese Medicine Hospital of Ningbo, Ningbo, China; ^3^Department of Neurosurgery, The Traditional Chinese Medicine Hospital of Linping District, Hangzhou, China

**Keywords:** ischemia stroke, inflammation, peripheral organs, brain, therapies

## Abstract

Stroke is a devastating disease with high morbidity, disability, and mortality, among which ischemic stroke is more common. However, there is still a lack of effective methods to improve the prognosis and reduce the incidence of its complications. At present, there is evidence that peripheral organs are involved in the inflammatory response after stroke. Moreover, the interaction between central and peripheral inflammation includes the activation of resident and peripheral immune cells, as well as the activation of inflammation-related signaling pathways, which all play an important role in the pathophysiology of stroke. In this review, we discuss the mechanisms of inflammatory response after ischemic stroke, as well as the interactions through circulatory pathways between peripheral organs (such as the gut, heart, lung and spleen) and the brain to mediate and regulate inflammation after ischemic stroke. We also propose the potential role of meningeal lymphatic vessels (MLVs)-cervical lymph nodes (CLNs) as a brain-peripheral crosstalk lymphatic pathway in ischemic stroke. In addition, we also summarize the mechanisms of anti-inflammatory drugs in the treatment of ischemic stroke.

## 1 Introduction

Stroke is the second leading cause of death and the third leading cause of adult disability worldwide (Sacco et al., 2013; Mozaffarian et al., 2015). According to the results of radiological examination, stroke is classified as ischemic and hemorrhagic. The majority of strokes are ischemic, primarily due to arterial thrombosis or cardiogenic embolism. Hemorrhagic stroke includes cerebral hemorrhage and subarachnoid hemorrhage and is often caused by rupture of a cerebral artery or an intracranial aneurysm (Campbell and Khatri, 2020). Stroke can lead to central and systemic inflammatory responses (DeLong et al., 2022; Simats and Liesz, 2022; Ohashi et al., 2023). Inflammation plays an important role in the pathophysiological process of both ischemic stroke and hemorrhagic stroke, which can affect the clinical outcome of stroke patients (DeLong et al., 2022; Ohashi et al., 2023). In this review, we will discuss the inflammatory response after ischemic stroke. In the acute phase of ischemic stroke, resident immune cells (such as microglia and astrocytes) in the brain are activated, and then circulating immune cells (such as neutrophils, lymphocytes, and monocytes/macrophages) can cross the damaged blood–brain barrier (BBB) and invade the lesion (Jayaraj et al., 2019; Lambertsen et al., 2019; Nakamura and Shichita, 2019; Huang et al., 2020). Resident and infiltrating immune cells jointly coordinate the post-stroke inflammatory response and communicate with each other through cytokines and signaling pathways (Xu S. et al., 2020; Zhang Z. et al., 2022). Therefore, inflammation plays an important role in the pathophysiological mechanism after ischemic stroke.

Recently, it has been found that the interaction between brain and peripheral organs plays a key role in the occurrence and progression of diseases. Among them, the most famous is the brain-gut axis, which is related to inflammatory bowel disease (IBD), Parkinson’s disease (PD), stroke and other inflammation-related diseases (Bonaz and Bernstein, 2013; Pluta et al., 2021; Honarpisheh et al., 2022; Tan et al., 2022). Recent studies also found that the crosstalk between meningeal lymphatic vessels (MLVs) and cervical lymph nodes (CLNs) is also an important pathway of brain-periphery interaction (Louveau et al., 2015; Ma Q. et al., 2017; Louveau et al., 2018; Ahn et al., 2019; Hu et al., 2020). In this review, we discuss the role of brain-peripheral crosstalk in inflammation after ischemic stroke and summarize the potential role of anti-inflammatory drugs in the treatment of ischemic stroke.

## 2 Inflammation in the pathophysiology of ischemic stroke

### 2.1 Inflammatory activation pathways after ischemic stroke

Although the damage mechanisms of injury are different in both ischemic and hemorrhagic stroke, the release of damage-associated molecular patterns (DAMPs) follows similar pathways. These DAMPs can be recognized by inflammatory cells to activate the sterile immune inflammatory response in and outside the brain, which play a vital role in increasing cellular death. Common DAMPs include high-mobility-group box 1 (HMGB1), peroxiredoxins (PRXs), adenosine 5′-triphosphate (ATP), DNA, and RNA (Shichita et al., 2017; Gulke et al., 2018; Di Virgilio et al., 2020). When the blood–brain barrier (BBB) is impaired after a stroke, immune cells can easily enter the central nervous system (CNS) from the blood (Huang et al., 2020). The explosive activation of local immune cells promotes the invasion of peripheral immune cells. The accumulation of immune cells provides significant conditions for the occurrence of inflammation. Pattern recognition receptors (PRRs) on inflammatory cells recognize DAMPs, which activate immune cells through multiple signaling pathways. Subsequently, immune cells gradually release cytokines, which then attract and activate more immune cells through chemotaxis, forming a positive feedback loop (Kim et al., 2019; Nakamura and Shichita, 2019; Gong et al., 2020; Maehara et al., 2021). Nowadays, there are mainly five types of PRRs have been described: Toll-like receptors (TLRs), C-type lectin receptors (CLRs), NOD-like receptors (NLRs), RIG-I-like receptors (RLRs), and Cytoplasmic DNA sensors (CDSs) (Gong et al., 2020). Once activated, PRRs initiate various innate immune signaling pathways, leading to the production of pro-inflammatory cytokines and type I interferons (IFN-I) (Gong et al., 2020). New evidence suggests that PRRs can be activated by endogenous DAMPs, resulting in cellular senescence and various human diseases (Roers et al., 2016; Li and Chen, 2018; Mangan et al., 2018).

### 2.2 Immune cells activation after ischemic stroke

Studies have shown that peripheral immune cells and resident glial cells could play an essential role in the post-stroke immune response (Xu S. et al., 2020; Zhang Z. et al., 2022). After ischemic stroke, immune cells in the brain such as microglia and astrocytes are immediately activated in response to ischemic injury. Subsequently, peripheral immune cells are activated and recruited to the brain to assist in the immune response (Chu et al., 2014; Jones et al., 2018). We will give an introduction to these immune cells.

Evidence indicated that microglia and astrocytes, resident innate immune cells in the brain tissue, exert both beneficial and detrimental effects after ischemic stroke (Qin et al., 2019; Patabendige et al., 2021). DAMPs activate microglia and astrocytes through multiple pathways after ischemic stroke. The activated microglia have two distinct functional phenotypes. M1-type (classical) microglia produce pro-inflammatory mediators, including interleukin (IL)-1β, interferon-gamma (IFN-γ), tumor necrosis factor alpha (TNF-α), IL-6, inducible nitric oxide synthase (iNOS), and proteases (MMP9, MMP3) (Yenari et al., 2010). Conversely, M2-type (alternative) microglia are characterized by the production of IL-10, transforming growth factor b (TGF-b), insulin—like growth factor, and vascular endothelial growth factor (VEGF), which are pro-angiogenic and anti-inflammatory (Ponomarev et al., 2013). It’s worth noting that this binary classification is oversimplified because microglia exist in many overlapping functional states. Microglia can either promote damage or facilitate repair, depending on the activation signals they receive (Ma Y. et al., 2017; Al Mamun et al., 2018). The dominant functional phenotype will change during the diverse period of stroke. (Jian et al., 2019; Qin et al., 2019; Ma et al., 2021). Cerebral hypoperfusion induces microglial activation, production of associated pro-inflammatory cytokines, and priming of microglial polarization toward the M1 phenotype, while the immune modulator fingolimod attenuates microglia-mediated neuroinflammation after white-matter ischemia and promotes oligoden-drocytogenesis by shifting microglia toward M2 polarization (Qin et al., 2017). Thus, shifting the phenotypic balance towards a restorative phenotype could be a novel therapeutic intervention for ischemic stroke.

After ischemic stroke, astrocytes perform multiple functions, which can be both harmful and beneficial to neuronal survival in the acute phase (Liu and Chopp, 2016). Astrocytic inflammatory responses to stroke may exacerbate ischemic lesions, yet astrocytes can also limit lesion expansion through anti-excitotoxic effects and release neurotrophic factors, thus providing neuroprotection (Markiewicz and Lukomska, 2006; Li et al., 2008; Cekanaviciute et al., 2014). Similarly, in the late recovery phase after stroke, glial scars may impede axonal regeneration, leading to diminished functional outcomes (Gris et al., 2007). However, astrocytes also contribute to angiogenesis, neurogenesis, synaptogenesis, and axonal remodeling, thereby promoting recovery of neurological function (Mauch et al., 2001; Wang et al., 2011, 2014). Therefore, the role of activated astrocytes following ischemic stroke is also a double-edged sword.

Neutrophils are the first peripheral immune cells to enter the brain after ischemic stroke. After activation, neutrophils produce cytokines to recruit other immune cells, engage in receptor-mediated phagocytosis to engulf microbes, and further release granular antimicrobial molecules as well as form neutrophil extracellular traps (NETs) (Amulic et al., 2012). Neutrophils can convert into N1 and N2 subtypes in response to external stimuli. The N1 can secrete pro-inflammatory factors and proteases to aggravate ischemic brain damage, while the N2 may have neuroprotective effects (Kang et al., 2020; Xie et al., 2022). The role of different neutrophil subsets in other points of neuroplasticity remains unclear.

Monocytes and macrophages are mononuclear phagocytes derived from macrophage/dendritic cell progenitors in the bone marrow (BM). During disease progression, they can enter the circulation in a C-C chemokine receptor 2 (CCR2)-dependent manner and migrate into tissues to generate tissue-resident macrophages termed monocyte-derived macrophages (MDMs), which express high levels of CD68.

Previous studies have shown that the number of monocytes peaks at Day 3 after ischemic stroke, and they differentiate into MDM (Fang et al., 2018). During ischemic stroke, dying/dead neurons release DAMPs, such as ATP, HMGB1, damaged DNA and peroxiredoxin family proteins, which can be recognized by PRRs, including Toll-like receptor TLR-2 and TLR-4, expressed by some innate immune cells, such as monocytes and macrophages (MMs), microglia and neutrophils (Shichita et al., 2012). Many studies have shown that activated MMs can polarize into distinct subtypes, including the well-known M1 and M2 subpopulations. The M1 subtype secretes proinflammatory cytokines, such as tumor necrosis factor alpha (TNF-α), interleukin (IL)-1β, IL-12, and IL-6, and can be distinguished by cell surface markers CD16 and CD32. The M2 phenotype produces TGF-β, IL-4, IL-10, and IL-13, and expresses CD206 and Arg1. The activation of MM subpopulations and other innate immune cells leads to neuroinflammation (Wattananit et al., 2016; Fang et al., 2018), the role of which has been well characterized in the acute phase of ischemic brain injury (Qiu et al., 2021). During the subsequent 2 weeks, MMs gradually shift from the proinflammatory M1 phenotype to the alternatively activated M2 phenotype, facilitating the resolution of inflammation (Wattananit et al., 2016; Fang et al., 2018). Moreover, phagocytosis of dying cells is another important function of MMs, which is associated with functional recovery of ischemic brain (Xiong et al., 2016; Han et al., 2020). During ischemic stroke, the inflammatory and phagocytic actions mediated by microglia and macrophages undergo dynamic changes. The inflammatory and phagocytic responses of microglia and macrophages vary over time in the acute, subacute, and chronic stages of ischemic stroke, and also vary depending on the location within the ischemic core or peri-infarct region (Chen et al., 2022). Additionally, although both M1 and M2 phenotypes express phagocytic receptors, they possess different phagocytic capabilities. M2 phenotype is more efficient in clearing dead cells than M1 phenotype (Kapellos et al., 2016). Currently, it remains unclear how the inflammatory phenotype and phagocytic capacity of microglia and macrophages cross-regulate, and the detailed molecular mechanisms and their impact on functional recovery after ischemic stroke. Therefore, future research needs to consider the inflammatory phenotype and phagocytic actions of microglia and macrophages in the context of time and location to optimize therapies for improving functional recovery.

T cells are key participants in cellular adaptive immunity and play roles in various neurological disorders. According to previous reports, the peak of T cell infiltration occurs at different time points. Some studies indicate peak infiltration within 24 h, while others show it around day 3 to day 7, and some during the chronic phase. This variability may be due to different stroke models and testing methods (Grønberg et al., 2013; Chu et al., 2014). The role of different T-cell subsets in ischemic stroke in the acute damage phase remains controversial (Zhang D. et al., 2021). The mode of CD4^+^ T-cell differentiation in response to brain injury ultimately determines stroke outcome. IFN-γ released from Th1 cells appears to either worsen outcomes or have an effect on brain infarct volume (Yilmaz et al., 2006; Shichita et al., 2009). The absence of IL-4 or neutralization of IL-4 (the main cytokine released by Th2 cells) can exert neuroprotective effects (Zhang et al., 2018). Similar to CD4^+^ T cells, CD8^+^ T cells also persist in the injured brain for weeks. The role of CD8^+^ T cells after ischemia is also controversial. The depletion of CD8^+^ T cells beginning 10 days post-tMCAO improved motor recovery (Selvaraj et al., 2021). However, Cai et al. (2022) discovered a new CD8^+^ T regulatory-like cells that could reprogram to upregulate leukemia inhibitory factor (LIF) receptor, epidermal growth factor–like transforming growth factor (ETGF), and interleukin 10 (IL-10) to exert neuroprotection and promoted long-term neurological recovery. The role of T cells after stroke may vary depending on their subsets and the time window post-stroke.

B cells were initially identified through research on antibody-producing cells. As key participants in humoral immunity, B cells promote immune responses through antigen presentation, antibody production, and cytokine secretion (Jain and Yong, 2022). The role of B cells during the acute phase of ischemic stroke remains inconclusive. Some studies have found no effect on infarct size and stroke outcomes (Schuhmann et al., 2017), while others have observed beneficial effects of B cells (Offner and Hurn, 2012). Considering that the adaptive immune response specific to central nervous system antigens occurs later than the innate immune response, the role of B cells in the subacute and chronic phases of ischemic stroke has drawn the attention of some researchers. Ischemic injury induces significant bilateral infiltration of B cells into remote brain regions, where they regulate motor and cognitive function by supporting neuronal vitality and dendritic branching (Ortega et al., 2020). In a distal middle cerebral artery occlusion (dMCAO) model, it was found that B cells infiltrate the infarct area in the chronic phase after stroke and secrete IgA and IgG, which may directly affect post-stroke cognition (Doyle et al., 2015).

In summary, both resident immune cells within the brain and infiltrating immune cells from the periphery are markedly activated after ischemic stroke. Given the dual role of immune cells, gaining a deeper understanding of the dynamic functional changes of various immune cell populations throughout the disease course and seeking effective interventions to modulate immune cell subtype transitions will provide new theoretical foundations for immunotherapy in ischemic stroke. Importantly, interactions of peripheral immune cells and brain resident cells are essential for brain injury and recovery. They play an essential role in the brain-peripheral crosstalk in inflammation after ischemic stroke (Muhammad et al., 2021; Zhang Z. et al., 2022).

## 3 Inflammation in brain-peripheral crosstalk after ischemic stroke

The brain is the command post for all systems and organs of the body. Once something goes wrong, such as a stroke, it can affect organs in multiple systems throughout the body. After a stroke, inflammatory response occurs in the CNS. The dysfunction of CNS and neuroinflammation can cause systemic inflammation (Wu et al., 2022). In the same way, peripheral inflammation can also impact the CNS through some pathways to induce brain injury (Liu Q. et al., 2019; Loppi et al., 2021). The review will discuss the mechanisms of brain-peripheral crosstalk in inflammation after ischemic stroke.

### 3.1 Inflammation and brain-gut axis in ischemic stroke

#### 3.1.1 The association between inflammation and brain-gut axis

Although the communications between the gut and the brain have been recognized for decades, the brain-gut axis has become an emerging research hotspot. Researchers found that bidirectional interactions of inflammatory signals between the intestine and the brain occur through three pathways. They are humoral pathway, cellular immune pathway and neuronal pathway (Agirman et al., 2021).

In humoral pathway, intestinal inflammation can disrupt the intestinal barrier and cause intestinal inflammatory mediators to enter the blood through the damaged intestinal barrier, as well as affect BBB permeability and then enter the CNS to induce neuroinflammation (Parker et al., 2020; Mou et al., 2022). Gut microbiota also plays a critical role in the mechanism of brain-gut axis. Data have demonstrated that microbial diversity collapses in the days following ischemic stroke onset (Winek et al., 2016a). Microglia were major effectors of inflammatory injury encountered after stroke (Singh et al., 2016). Gut microbiota control maturation and function of microglia throughout life through short-chain fatty acids (SCFAs) production, vagal transit, or by production of other metabolites that cross the BBB. Microbial tryptophan metabolites modulate microglial and astrocytic activation in an arylhydrocarbon receptor-dependent manner (Lee et al., 2015), such as modulation of TGFα and VEGFβ. In addition to crossing BBB, SCFAs seem to play an important role in maintaining its integrity, which is tightly associated with controlled passage of molecules and nutrients from the circulation to the brain, playing a central role in brain development and the preservation of CNS homeostasis (Braniste et al., 2014). Additionally, the hypothalamic–pituitary–adrenal (HPA) axis is activated to release glucocorticoids, which can regulate intestinal function (Agirman et al., 2021).

In cellular immune pathway, immune cells are identified in the vicinity of the meningeal venous sinuses, and people also have found that these cells were derived from the gut through sequencing and can release cytokines or immunoglobulins to regulate neuroinflammation. But the mechanism of inducing immune cells migration remains undefined (Fitzpatrick et al., 2020; Sanmarco et al., 2021). Some people suppose that the stress response after CNS injury can change the composition of microbiota, which would stimulate migration of intestinal immune cells to the CNS (Cryan et al., 2019). Some microbiota metabolites can also regulate the development and function of resident immune cells in the CNS (Rudzki and Maes, 2020; Mossad et al., 2022).

In neuronal pathway, neurons that connect the CNS to the gut can transmit signals bidirectionally (Agirman et al., 2021). Gut-derived inflammatory stimuli are transmitted to the CNS through the afferent fibers of the vagus nerve and the signal may activate multiple neural circuits. After the advanced CNS analysis, efferent fibers transmit signals to intestinal immune cell to limit the invasion of harmful microorganisms and promote the growth of probiotics to suppress intestinal inflammation (Fulling et al., 2019; Han et al., 2022).

Several inflammation-associated diseases are related to brain-gut axis. In inflammatory bowel disease (IBD), there is a bidirectional effect between psychological health and IBD progression (Gracie et al., 2018). Psychological disorders may activate the hypothalamic–pituitary-adrenocortical axis and sympathetic-adrenal medulla system to increase the secretion of glucocorticoids and catecholamines to regulate gastrointestinal function. In turn, inflammatory activity in the gut can also influence the CNS by increasing intestinal permeability, changing the composition of intestinal microbes and stimulating the vagus nerve, which can cause psychological disorders (Brzozowski et al., 2016; Gracie et al., 2018, 2019). Additionally, depressive symptoms can be alleviated by improved dietary structure and supplementation with probiotics (Reininghaus et al., 2020; Marx et al., 2021). Inflammation also plays a vital role in brain-gut crosstalk in neurodegenerative diseases. Gut dysbiosis and intestinal inflammation can facilitate inflammatory factors to enter the circulation through the highly permeable intestinal wall, which causes BBB damage and neuroinflammation to promote the occurrence and progression of Alzheimer’s disease (AD) and Parkinson’s disease (PD) (Doifode et al., 2021; Tan et al., 2022). Animal studies have also found that fecal transplantation and supplementation of specific metabolites could improve the motor symptoms of PD mice by relieving intestinal inflammation while inhibiting neuroinflammation in the substantia nigra (Sampson et al., 2016; Perez-Pardo et al., 2019; Zhao et al., 2021). Fecal transplantation and probiotic intervention are also considered be new treatment of AD (Doifode et al., 2021). In multiple sclerosis (MS), specific microorganisms in the gut and their metabolite can induce peripheral CD4^+^ T cells to be polarized into pro-inflammatory Th1 cells and Th17 cells or anti-inflammatory regulatory T cells. These T cells can migrate to the CNS through circulation and regulate neuroinflammation to affect the progression of MS (Kadowaki and Quintana, 2020; Ghezzi et al., 2021). With the involvement of gut microbes, mice fed a high-fat diet will activate microglia to develop hypothalamic inflammation (Valdearcos et al., 2014). Neuroinflammation can lead to leptin resistance and feeding behavior disorder to induce obesity, which implies that obesity is also associated with inflammatory signaling in the brain-gut axis (Valdearcos et al., 2014; Torres-Fuentes et al., 2017; Heiss et al., 2021). Targeting the inflammatory signals of the gut-brain axis will probably become an effective therapy for these inflammation-related diseases.

#### 3.1.2 The role of brain-gut axis in inflammation after ischemic stroke

**Figure 1:**
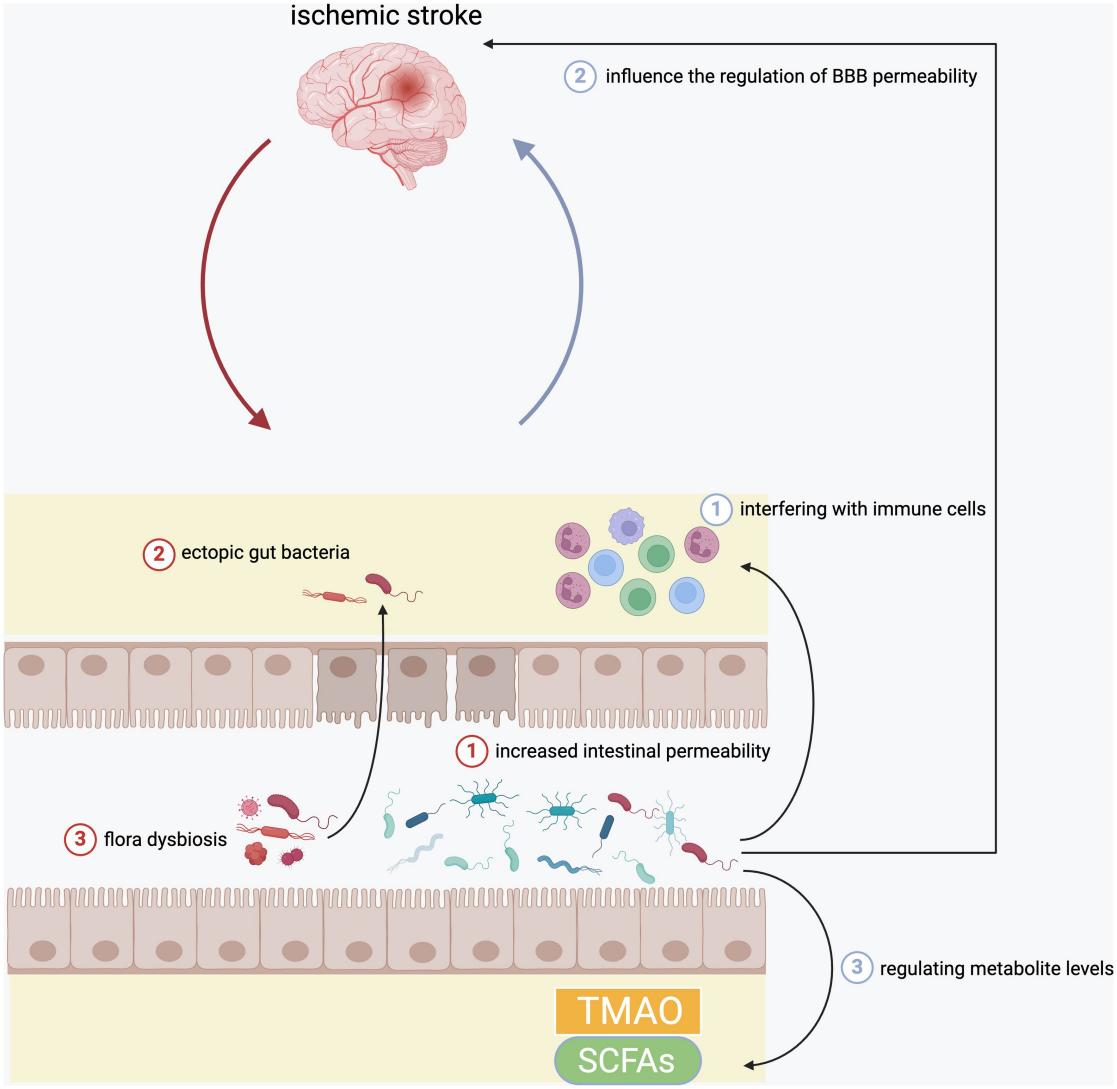
The interaction between brain and gut in the inflammation after ischemic stroke. Ischemic stroke would damage intestinal epithelial cells to increase the intestinal permeability, which further aggravated the translocation of intestinal bacteria. In addition, it could also cause flora dysbiosis. In turn, gut microbiota could also affect ischemia brain through regulating circulating immune cells, BBB permeability, as well as serum levels of TMAO and SCFAs.

In recent years, a large number of studies verify the relationship between inflammation after ischemic stroke and brain-gut axis, in which gut microbiota is the most critical player. The following will separately introduce the bidirectional influence between the brain and the gut after a stroke (Figure 1).

The gut is the largest microbial reservoir in the human body, and these microbes contain far more genes than the human genome (Gilbert et al., 2018). Many animal experiments have found that the gut microbiota in the mice stroke model has undergone significant changes (Crapser et al., 2016; Stanley et al., 2018; Xu et al., 2021). Crapser et al. found that after transient middle cerebral artery occlusion (MCAO), mice have increased intestinal permeability and ectopic gut bacteria, and only young mice could cope with the infection caused by these ectopic bacteria, while old mice were more likely to develop sepsis (Crapser et al., 2016). Stanley et al. also found that stroke caused significant changes in the microbial composition of the intestinal mucosa in mice. There was an increased abundance of *Akkermansia muciniphila* and an excessive abundance of clostridial species after stroke (Stanley et al., 2018). Xu et al. also found that acute ischemic stroke (AIS) could cause mice intestinal ischemia during a short period of time, which would produce excessive nitrate in the gut to lead to intestinal dysbiosis (Xu et al., 2021). At present, more than one clinical study has found that there was specific upregulated or downregulated gut microbiota in stroke patients (Benakis et al., 2020; Peh et al., 2022). Some studies illustrated the same conclusion that there showed a higher abundance in Streptococcus, Lactobacillus and Escherichia, and a lower abundance in Eubacterium, Roseburia in stroke patients compared to healthy participants (Peh et al., 2022). In addition to changes in the intestinal microbiota, stroke can also cause cecal dysbiosis, loss of goblet cells and decreased mucus (Houlden et al., 2016).

The gut microbiota can also manipulate the inflammation state of the brain and the severity of cerebral infarction in some ways (Peh et al., 2022). One study found that cerebral ischemia induces an increasing abundance of Enterobacteriaceae. In turn, these harmful bacteria aggravate the neuroinflammation of the ischemic brain by enhancing systemic inflammation and aggravate the degree of cerebral infarction (Xu et al., 2021). Benakis et al. showed that the change of intestinal flora caused by antibiotics improved the severity of brain damage in ischemic stroke. Changes in the intestinal flora lead to an increase in regulatory T cells and a decrease in interleukin-17-positive γδ T cells, which inhibits the transport of effector T cells to the leptomeninges after stroke (Benakis et al., 2016). Winek et al. found that extensive depletion of the gut microbiota could reduce survival in experimental stroke mice and exhibited systemic immunosuppression on day 5 after cerebral ischemia. However, continuous preventative antibiotic treatment or restoration of commensal microbiota in microbiota depleted mice could improve the situation (Winek et al., 2016b). Another study demonstrated that normal gut microbiota could reduce BBB permeability in mice by up-regulating the expression of tight junction proteins, which implied that the changes of gut microbiota could influence the regulation of BBB permeability after stroke to affect the progression of neuroinflammation (Braniste et al., 2014). The alteration of gut microbiota can cause changes in the levels of some important metabolites to affect the brain. Trimethylamine (TMA) is produced by the action of TMA cleaving enzymes in the gut microbiome. TMA is then further oxidized to trimethylamine N-oxide (TMAO) (Nam, 2019). TMAO can promote atherosclerosis and thrombosis through promoting macrophage-derived foam cell formation, enhancing platelet reactivity, altering bile acid and cholesterol transport, and activating inflammatory pathways (Peh et al., 2022). Studies have also found that high levels of TMAO can increase the risk of stroke and the size of cerebral infarction. And changes in gut bacteria cause up-regulation or down-regulation of TMAO levels to affect the risk and severity of ischemic stroke (Nam, 2019; Zhu et al., 2021). Other important metabolites of gut microbiota, short-chain fatty acids (SCFAs), have the positive effect on ischemic brain (Peh et al., 2022). Animal studies have shown that transplantation of SCFAs-producing bacteria increased the levels of SCFAs in serum, gut and brain to repair the intestinal barrier, inhibit inflammation and improve neurological dysfunction (Chen et al., 2019; Lee et al., 2020). In addition to affecting the brain, the imbalance of the intestinal microbiota after a stroke can also cause infections in various organs of the body, such as pneumonia. These infections also affect the prognosis of stroke (Stanley et al., 2016). In the future, it might become a new means to treat stroke to restore the normal intestinal microbial composition and supplement beneficial metabolites.

### 3.2 Inflammation and brain-heart axis in ischemic stroke

Cardiovascular complications are the second leading cause of death after stroke (Chen et al., 2017). The most common cardiac complications include cardiac arrhythmias, myocardial infarction, congestive heart failure, neurogenic stress cardiomyopathy (NSC), and Takotsubo cardiomyopathy. Additionally, multiple cardiac diseases and AIS share the same risk factors. Thus, it will be necessary to research the brain-heart syndrome and there is even a neurocardiology major (Samuels, 2007; Battaglini et al., 2020). The mechanisms for brain-heart interactions after stroke are complex, which include activation of the HPA axis, enhanced sympathetic and parasympathetic activity, catecholamine release, gut dysbiosis and inflammation (Chen et al., 2017). The following will discuss the role of inflammatory signaling in the brain-cardiac axis after stroke.

Disruption of the BBB is a vital alteration of the pathophysiological cascade after ischemic stroke, which plays an important role in the progression of systemic inflammation (Chen et al., 2017; Varatharaj and Galea, 2017; Galea, 2021). After a stroke, in addition to the immune response of resident immune cells in the brain, peripheral macrophages and neutrophils also infiltrate into lesions through the damaged BBB because of chemotaxis. This not only enhances the local inflammatory response in the ischemic brain but also further aggravates the BBB damage (Battaglini et al., 2020; Scheitz et al., 2022; Ziaka and Exadaktylos, 2023). This is a vicious cycle. Disruption of the BBB promotes the entry of inflammatory cells and factors into the brain, while local inflammation increases BBB permeability. The severe damage of BBB may facilitate inflammatory factors and immune cells in the inflammatory response area as well as brain-derived antigens from injured brain cells into the circulation, which lead to the inflammatory response of the heart (Battaglini et al., 2020; Scheitz et al., 2022; Ziaka and Exadaktylos, 2023). In addition, the brain-gut axis plays a mediating role in the brain-heart axis. Intestinal barrier damage and flora dysbiosis after a stroke cause intestinal bacterial translocation and endotoxin release, which further aggravates systemic inflammation to lead to myocardial injury (Chen et al., 2017; Battaglini et al., 2020). TMAO, a metabolite derived from gut microbes, also adversely affects cardiomyocytes and coronary endothelial cells (Peh et al., 2022). An animal study illustrated that the number of granulocytes in myocardial tissue increased, and the concentrations of proinflammatory cytokines IL-1β and IL-6 were also nearly doubled in mice after transient middle cerebral artery occlusion (tMCAO), which could lead to cardiac dysfunction and hemodynamic impairment (Vornholz et al., 2021). Hermanns et al. found that in middle cerebral artery occlusion (MCAO) mice, the severity of neuroinflammation correlated with cardiac function. Sustained neuroinflammation was associated with a further decline in left ventricular systolic function. In the process of stroke progression, inhibition of microglia activation could also affect cardiac systolic function (Hermanns et al., 2022). Additionally, studies demonstrated that stroke not only damaged the contractility of the heart, but also made the myocardium more sensitive to ischemia (Meloux et al., 2018). Neuroinflammation after ischemic stroke can affect the heart, but the molecular mechanisms induced myocardial changes remain to be determined. In patients after acute ischemic stroke, researchers also detected significantly increased levels of biomarkers of myocardial injury, such as cardiac troponin (cTn), brain natriuretic peptide (BNP) (Agewall et al., 2011; Xu C. et al., 2020; Scheitz et al., 2021). The most easily observed after a stroke is the tachycardia caused by sympathetic excitement. Studies showed that there is a crosstalk between sympathetic excitement and inflammation (Winklewski et al., 2014; Chen et al., 2017). After an ischemic stroke, damaged neurons and glial cells stimulate the hypothalamus through releasing inflammatory factors to induce the intense activation of sympathetic nervous system and the release of catecholamines. These can regulate the number and function of lymphocytes and monocytes to affect cardiomyocyte inflammation and myocardial injury (Kenney and Ganta, 2014; Winklewski et al., 2014; Chen et al., 2017). In turn, hemodynamic changes caused by cardiac dysfunction will also impact cerebral perfusion to aggravate neuroinflammation in the ischemic lesions, which forms a vicious circle (Samuels, 2007; Chen et al., 2017; Battaglini et al., 2020). It will improve the prognosis of ischemic stroke and reduce the risk of death to further explore brain-heart syndrome.

### 3.3 Inflammation and brain-lung axis in ischemic stroke

Pulmonary dysfunction after brain injury is common. Patients with stroke often present with pulmonary complications such as pneumonia, pleural effusion, acute respiratory distress syndrome, pulmonary edema, and respiratory failure (Robba et al., 2019; Herpich and Rincon, 2020). The pathophysiological mechanism of acute lung injury in patients with severe brain injury (such as stroke and traumatic brain injury) is complex, but inflammatory response may play an important role in brain-lung crosstalk (Mascia, 2009).

Stroke can lead to cerebral and systemic inflammatory response (Xiong et al., 2016; Han et al., 2020; Muhammad et al., 2021; Xie et al., 2022). Inflammation can promote neutrophils and activated macrophages to migrate into the alveolar space as well as cause the damage to alveolar type II epithelial cells (Robba et al., 2019). Samary et al. found that the damage to type 2 pneumocytes and endothelial cells, inflammatory cell infiltration and decreased phagocytic capacity of alveolar macrophages in the lung of rats with focal ischemic stroke. And the levels of proinflammatory mediators in brain, lung, and plasma all increased (Samary et al., 2018). Another animal experiment showed that the dysregulation of hepatocyte growth factor (HGF), transforming growth factor-α (TGF-α), and C-C motif chemokine ligand 2 (CCL2) were the characterization of lung injury in MCAO mice. These proteins all had the function of regulating inflammation (Faura et al., 2022). In turn, lung injury can also affect the brain through the autonomic nervous system and immune pathways, which aggravate the secondary brain injury after ischemic stroke (Robba et al., 2019). The role of brain-lung crosstalk in stroke is unquestionable, but the molecular mechanisms of brain-lung axis need to be further explored, which will help in the management of assisted ventilation in patients with severe stroke.

### 3.4 Inflammation and brain-spleen axis in ischemic stroke

Spleen is an important immune organ in human body. In recent years, people begin to focus on the role of spleen activation in inflammation and immune response after an ischemic stroke (Han et al., 2021). Regarding the connection between brain and spleen, the spleen is almost always innervated by sympathetic nerve fibers. Norepinephrine is the main neurotransmitter, and no evidence of cholinergic innervation has been found (Verlinden et al., 2019). A large number of animal experiments and clinical experiments proved that the spleen volume decreased significantly in the acute phase of ischemic stroke (Han et al., 2021). A human experiment proved that the spleen would shrink to its smallest size within 1 day after an ischemic stroke. The state would continue until the third day after the stroke, and then the spleen would gradually increase in size until it returned to its original size (Sahota et al., 2013). In an animal experiment, the size of the spleen was significantly reduced 24 to 48 h after MCAO in rats. After 96 h, the volume of the spleen recovered to be no statistically different from that of the sham-operated rats (Seifert et al., 2012a). The sharp reduction in volume is mainly due to the activation of sympathetic nervous system after a stroke, which releases a large amount of catecholamines, which act on the α1-adrenergic receptor (α1-AR) of the spleen (Shephard, 2016; Han et al., 2021). The relationship between the change of spleen volume and the size of infarct is still controversial (Han et al., 2021). An animal experiment showed that the size of the spleen had nothing to do with the infarct size (Chiu et al., 2016). But more researchers agreed that the spleen volume was negatively correlated with the infarct size--the more severe the spleen atrophy, the larger the infarct size (Han et al., 2021). Severe atrophy of spleen in stroke patients were associated with poor prognosis (Sahota et al., 2013). And a large number of studies indicated that splenectomy before stroke could reduce the size of infarction and inhibited neuroinflammation (Ajmo et al., 2008; Seifert et al., 2012b; Chauhan et al., 2018; Seifert and Offner, 2018; Sternak et al., 2022). Although the blood volume of the spleen is not high, the spleen is the largest immune organ in the body, and its reduction in size will inevitably lead to a large number of immune cells overflowing (Liu et al., 2015). The overflowing immune cells are activated. New antigens that activate immune cells in the spleen include microtubule-associated protein 2 (MAP 2), N-methyl-D-aspartate receptor subunit 2 (NR-2A), myelin basic protein (MBP) and myelin oligodendrocyte glycoprotein (MOG) (Becker, 2009; Miro-Mur et al., 2016). Activated immune cells are recruited by chemokines CC and CXC released from ischemic brain to cross the impaired BBB and enter the CNS from the spleen mainly through the CCL2-CCR2 signaling axis, causing adverse effects (Mirabelli-Badenier et al., 2011).

In this vicious cycle after ischemic stroke, sympathetic nerves are activated to release catecholamines, causing the decrease in spleen volume. Subsequently, immune cells and cytokines are released into the circulation from the spleen. These immune cells are activated and recruited to migrate into the CNS by antigens and chemokines released from ischemic brain and aggravate the inflammatory response. Thus, modulation of the spleen is likely to be an effective strategy to suppress inflammation after ischemic stroke.

### 3.5 Systemic inflammatory response syndrome (SIRS) after ischemic stroke

SIRS is a systemic inflammatory state because of the response to stimuli (Singer et al., 2016). Studies showed that patients with more severe acute ischemic stroke have higher rates of SIRS (Pedersen et al., 2004; Boehme et al., 2013; DeLong et al., 2022). For example, the pneumonia mentioned above is actually the most common type of infection after stroke and the lung is also the first organ to be hit in SIRS (Winklewski et al., 2014; Elkind et al., 2020). The occurrence of SIRS has an important impact on clinical outcome (Elkind et al., 2020).

As mentioned above, stroke can lead to local and systemic inflammatory response, which need the brain and multiple organs of the whole body to interact with each other. This suggests that people not only focus on the brain, but also pay more attention to the whole body for the treatment and management of stroke, so as to further improve the prognosis of stroke patients.

### 3.6 Inflammatory signals disseminated through the lymphatic pathway in brain-peripheral crosstalk in ischemic stroke

Inflammation that is transmitted through circulation in brain-peripheral crosstalk has been described above. In addition, inflammatory signals disseminated through the lymphatic pathway have also received attention. It used to be widely believed that the brain lacked lymphatic vessels. In 2015, Louveau et al. discovered the structure of meningeal lymphatic vessels (MLVs) in the dura mater of mice and demonstrated that MLVs could drain CSF to peripheral cervical lymph nodes (CLNs) (Louveau et al., 2015). According to the anatomical location, MLVs can be divided into dorsal MLVs and basal MLVs (Ahn et al., 2019). Studies illustrated that the basal MLVs were mainly responsible for draining CSF, while the dorsal MLVs mainly mediated inflammatory factors and immune cells to enter CLNs (Ma Q. et al., 2017; Louveau et al., 2018; Ahn et al., 2019; Hu et al., 2020). MLVs are important structures that link the CNS to the peripheral immune system (Jacob et al., 2022). A large number of studies found that MLVs played a vital role in the occurrence, progression and prognosis of neurodegenerative diseases, traumatic brain injury, brain tumors and stroke (Da Mesquita et al., 2018; Rasmussen et al., 2018; Bolte et al., 2020; Song et al., 2020; Chen S. et al., 2021; Wang et al., 2023). Next, we will discuss the pathophysiological role of MLVs in the brain-peripheral crosstalk of inflammation in stroke.

**Figure 2:**
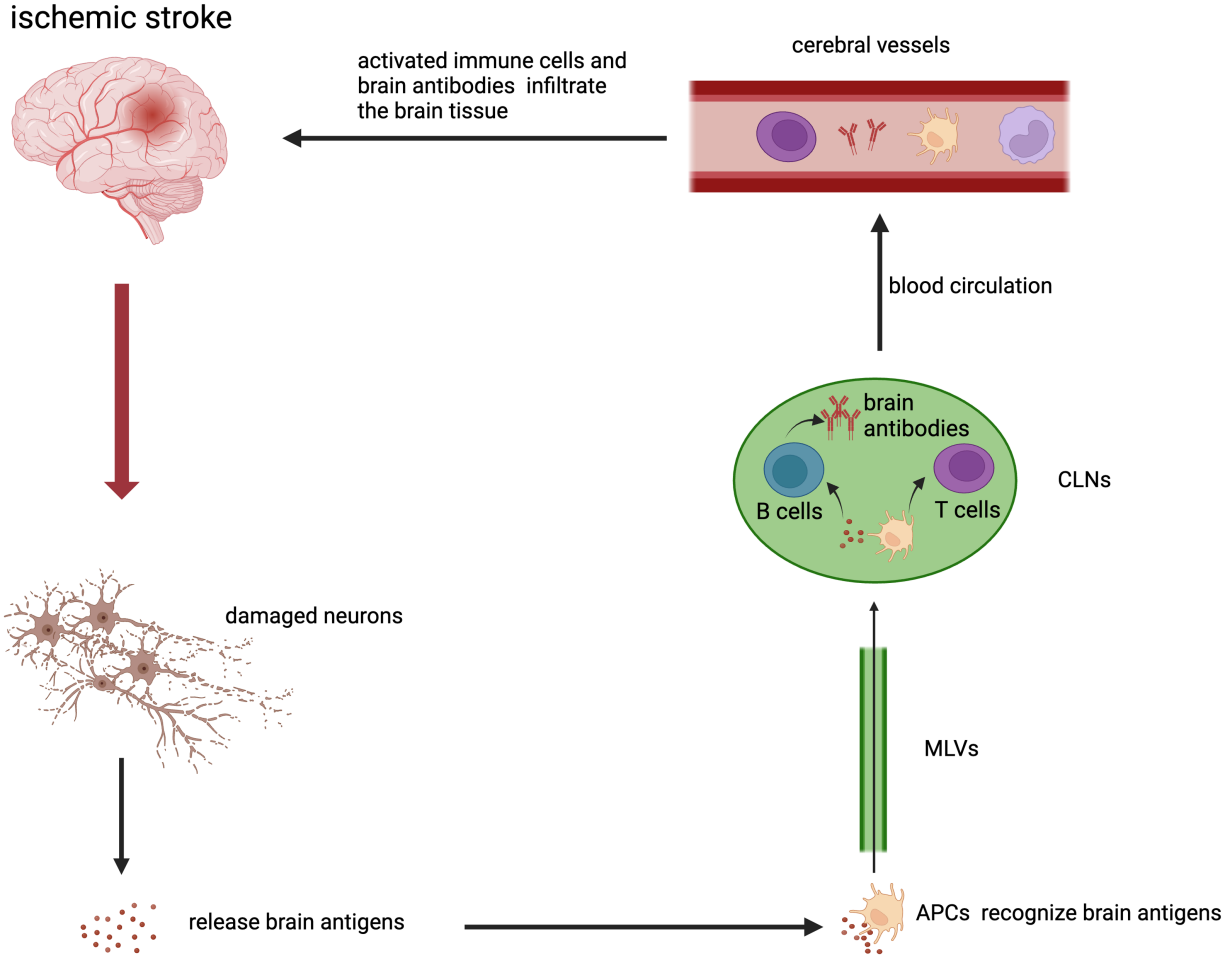
The brain-peripheral lymphatic crosstalk in the inflammation after ischemic stroke. Brain antigens were released from damaged neurons in the infarction area. APCs that bind and recognize brain antigens could migrate to peripheral CLNs through MLVs. APCs activated T cells and B cells in CLNs. Activated immune cells and brain antibodies could enter the cerebral vessels through the blood circulation and then infiltrate in the ischemic brain.

Meningeal lymphatics has been shown to be involved in the transport of macromolecules in the brain to CLNs (Aspelund et al., 2015). One study found an increase of brain-derived antigens in CLNs after acute stroke, which could activate immune cells in lymphoid tissues. CD68+ macrophages expressing MHC class II receptors and CD69+ T cells were the main cells in the immune response. And increased reactivity of lymphoid tissue to different classes of brain-derived antigens might be associated with better or worse clinical outcomes (Planas et al., 2012). Dendritic cell (DC) is an important antigen-presenting cells (APC) after stroke. DC can take up antigens and migrate to secondary lymphoid tissues. This promotes the generation of Tregs or effector T cells to induce immune tolerance or immune response (Miro-Mur et al., 2016). B cells can also produce corresponding brain antibodies (Chen J. et al., 2021). The molecules involved in DC migration to CLNs are mainly chemokine receptor CCR7 and its chemokine ligand CCL21 (Arasa et al., 2021). The CCR7-CCL21 axis is also an important pathway for T cells to migrate to CLNs (Louveau et al., 2018). Then, immune cells that are activated in CLNs can enter the circulation and infiltrate the lesion of the brain and exert proinflammatory or anti-inflammatory effects. VLA-4 and LFA-1 integrins or other molecules expressed by immune cells can contribute to migration (Schlager et al., 2016). In summary, cerebral ischemia causes cells death to releases brain antigens. Brain antigens are drained to the CLNs. APC can recognize brain antigens and migrate to the CLNs to activate T cells and B cells. Activated T cells, monocytes/macrophages and brain antibodies enter the blood circulation. Lastly, they infiltrate into the brain tissue from cerebral vessels (Figure 2).

An animal study showed that VEGFR3 was phosphorylated in CLNs after focal cerebral ischemia, which regulate lymphatic endothelial cell proliferation to promote macrophage proliferation and increased IL-β expression. Blocking VEGFR3 signaling and lymph node dissection can reduce the infiltration of pro-inflammatory macrophages in the ischemic area (Esposito et al., 2019). Another animal experiment demonstrated that VEGFR3 signaling controlled the development of MLVs (Yanev et al., 2020). Thus, VEGFR3 signaling play an important role in brain-peripheral lymphatic crosstalk. Toh et al. illustrated that patients with ischemic stroke would have lymphatic dysfunction and this disorder took some time to gradually recovers after the attack (Toh and Siow, 2021). Bai et al. showed that cranial bone transport (CBT) could improve the drainage of meningeal lymph and reduce the infiltration of T cells in MCAO rats, which relieved neuroinflammation and improved neurological deficits (Bai et al., 2022). Meningeal lymphatic hypoplasia also increased infarct volume of mice after tMCAO (Yanev et al., 2020). The conclusion of these studies suggested that maintaining the normal function and complete structure of MLVs might have a positive effect on ischemic stroke. Exploring the MLVs-CLNs axis will facilitate our understanding of the inflammatory response after stroke. It also provides a new potential therapeutic target.

## 4 Anti-inflammatory drugs in ischemic stroke

At present, it is generally believed that inflammation plays an important role in the pathogenesis of ischemic stroke, and the significance of anti-inflammatory drugs in the treatment and prevention of ischemic stroke has also attracted more and more attention (Kelly et al., 2021) (Table 1).

**Table 1:** Anti-inflammatory therapies in the treatment of ishemic stroke.

Anti-inflammatory therapy	Anti-inflammatory mechanism	References
Aspirin	Indirectly reduced release of inflammatory cytokines and ROS by inhibiting COX-1 to suppress platelets. Inhibition of granulocytes by triggering the formation of lipoxins. Increasing release of adenosine into extracellular fluid to scavenge hydroxyl radicals by regulating COX-2.	Russo et al., (2016), Montinari et al. (2019), Amann and Peskar (2002)
Statins	Decreased expression of adhesion molecules on the surface of immune cells and proinflammatory proteins. Decreased level of pro-inflammatory factors and CRP.	Vaughan and Delanty (1999), Schultz et al. (2019) Alikiaii et al. (2021)
Curcumin	Inhibition of the activation of microglia and the NF-κB signaling pathway as well as reduced release of IL-1β, IL-6 and TNF-α.	Bhat et al. (2019), Ullah et al. (2017)
Flavonoids	Inhibition of activation of TLR4/NF-κB signaling pathway and the expression of NLRP3 inflammasome and IL-1β as well as IL-6.	Martinez-Coria et al. (2023)
Melatonin	Reduced levels of TNF-α and IL-1β in the ischemic region. Inhibition of TLR4 inflammatory signaling pathway. Transformation of the phenotype of microglia through regulating STAT3 signaling pathway. Inhibition of the migration of neutrophils and macrophages/monocytes into the brain as well as the activation of microglia. Inhibition of MMP-9.	Rancan et al. (2018), Chen K. H. et al. (2020), Chumboatong et al. (2022), Liu Z. J. et al. (2019), Lee et al., (2007), Hung et al. (2008), Tai et al. (2010)
Probiotics/Prebiotics	Indirectly mitigation of inflammatory response in the brain and gut by maintaining intestinal barrier function and the levels of neuroprotective metabolites.	Yuan et al. (2021), Savigamin et al. (2022)
Polymyxin B	Regulation of gut microbiota and improvement of endotoxemia.	Kurita et al. (2020)
Lipopolysaccharide precondition	Inhibition of splenic atrophy, decreased levels of pro-inflammatory immune cells as well as increasing levels of regulatory B cells.	Wang et al., (2018)
Stem cell therapy	Inhibition of splenic atrophy, decreased levels of TNF-α and IFN-γ as well as regulation of T cells in the spleen.	Wang et al. (2019), Acosta et al. (2015)
Anti-CD147 antibody	Inhibition of monocyte/macrophage and reduced levels of TNF-α, IL-6 and IL-1β in the spleen.	Jin et al. (2019)
XPro1595 and etanercept (anti-TNF agents)	Regulation of the number of peripheral splenic T cells and inhibition of the infiltration of granulocytes into the brain.	Clausen et al. (2014)
Albumin	Inhibition of the expression of TLR4 and increased levels of regulatory T cells and IL-10 in the spleen.	Wang et al. (2013)
Cocaine and amphetamine regulated transcript (CART)	Regulation of CD4+/CD8+ ratio in the blood and spleen.	Chang et al. (2011)
Acetylcholine and nicotine (α7nAChRs agonists)	Inhibition of the synthesis and release of pro-inflammatory cytokines from macrophages in the spleen and gut.	Hoover (2017)

### 4.1 Common drugs with anti-inflammatory effects

Antiplatelet drugs and statins (HMG-CoA reductase inhibitors) are the most common drugs used for the prevention and treatment of ischemic stroke, and they have anti-inflammatory effects (Toyoda, 2009; Kelly et al., 2021). Aspirin is the first antiplatelet agent approved for secondary stroke prevention (Toyoda, 2009). For patients with non-cardioembolic ischemic stroke or transient ischemic attack (TIA), the use of low-dose aspirin (alone or in combination) can reduce the risk of recurrent stroke and other advent cardiovascular events (Russo et al., 2016). Low-dose aspirin can irreversibly inhibit cyclooxygenase-1 (COX-1) in platelet to lead to decreasing production of TXA_2_, which suppresses platelet aggregation and preventing thrombosis. When platelet activation in vascular lesions is inhibited, it may also play an indirect role, such as reducing the release of inflammatory cytokines and reactive oxygen species (ROS) (Russo et al., 2016; Montinari et al., 2019). In addition, the formation of aspirin-triggered lipoxins could inhibit the activity of granulocytes (Amann and Peskar, 2002). Sodium salicylate, the biotransformation product of aspirin, could regulate cyclooxygenase-2 (COX-2) expression, increase the release of adenosine into the extracellular fluid, and scavenge hydroxyl radicals (Amann and Peskar, 2002). These mechanisms are most likely also related to the anti-inflammatory effects of aspirin. Statins such as atorvastatin and rosuvastatin also play the positive role in the prevention and treatment of ischemic stroke (Vaughan and Delanty, 1999). In addition to lowering cholesterol levels, statins also have a variety of effects, such as improving endothelial function, antioxidant, anti-inflammatory, and anti-platelet effects (Cimino et al., 2007; Malfitano et al., 2014). Statins can reduce the bioavailability of isoprenoids, which may reduce the expression of adhesion molecules on the surface of immune cells and the production of proinflammatory proteins (Vaughan and Delanty, 1999). A clinical study showed that statins treatment before stroke could decrease the levels of pro-inflammatory factors after stroke, and RhoA GTPase as well as its downstream effectors might be the targets for statins to exert anti-inflammatory effects (Schultz et al., 2019). Alikiaii et al. also found that statins treatment could reduce the level of C-reactive protein (CRP) in stroke patients (Alikiaii et al., 2021).

Some compounds derived from plants can exert anti-inflammatory effects by inhibiting the production of ROS, regulating inflammatory factors and microglia (Chen H. et al., 2020; Tao et al., 2020). Studies demonstrated that the use of these drugs can reduce the neuroinflammatory cascade caused by cerebral ischemic injury (Tao et al., 2020). Curcumin, a polyphenolic compound, could inhibit the activation of microglia and the NF-κB signaling pathway to reduce the release of pro-inflammatory cytokines (IL-1β, IL-6 and TNF-α) and apoptosis (Ullah et al., 2017; Bhat et al., 2019). Thus, it could play a neuroprotective role in cerebral ischemia. Flavonoids also belong to the group of polyphenols. *In vitro* studies, flavonoids could inhibit the activation of the TLR4/NF-κB signaling pathway and the expression of NLRP3 inflammasome and proinflammatory cytokines such as IL-1β and IL-6. In animal experiments, flavonoids played an anti-inflammatory role and reduce infarct volume and neurological deficits (Martinez-Coria et al., 2023). Li et al. also suggested that polyphenols derived from natural plants could exert anti-inflammatory activities in ischemic stroke by regulating the TLR4/NF-κB pathway to regulate the polarization of microglia (Li R. et al., 2022).

Melatonin is also one of the potential neuroprotective agents. Melatonin is a neurohormone mainly synthesized and released by the pineal gland, which mainly regulates the circadian rhythm (Vasey et al., 2021). In cerebral ischemia, melatonin can also play a neuroprotective role through a variety of pathways (Ramos et al., 2017). We mainly discuss its anti-inflammatory effects in ischemic stroke. Rancan et al. found that melatonin treatment could reduce the levels of TNF-α and IL-1β in the ischemic region in aging rats with cerebral ischemia (Rancan et al., 2018). Chen et al. demonstrated that melatonin could inhibit TLR4-mediated inflammatory signaling pathway to exert anti-inflammatory effects (Chen K. H. et al., 2020). Agomelatine, a melatonin receptors agonist, suppressed the activation of microglia and reduced the NLRP3 inflammasome through TLR4/NLRP3 signaling pathway in MCAO rats (Chumboatong et al., 2022). Another animal experiment illustrated that melatonin could promote microglia to switch from pro-inflammatory to anti-inflammatory phenotype through regulating STAT3 signaling pathway (Liu Z. J. et al., 2019). In rats with transient focal cerebral ischemia, intravenous melatonin effectively reduced the migration of circulating neutrophils and macrophages/monocytes into the brain, and inhibited the activation of microglia in the lesion area (Lee et al., 2007). In addition, melatonin could inhibit the activation and expression of matrix metalloproteinase-9 (MMP-9) after cerebral ischemia, which would maintain the BBB integrity and reduce the infiltration of peripheral immune cells into lesion (Hung et al., 2008; Tai et al., 2010).

### 4.2 Anti-inflammatory therapies associated with brain-peripheral crosstalk

Melatonin and statins, already mentioned above, can exert anti-inflammatory effects indirectly through regulating the brain-gut axis. Melatonin has been shown to interact with gut microbiota (Ghareghani et al., 2018; Zhang Y. et al., 2022). Studies showed that regulating the composition of gut microbiota by supplementing prebiotics and other means could promote gut microbes to produce more melatonin or tryptophan metabolites, which might play an anti-inflammatory role in ischemic stroke (Li S. et al., 2022; Lian et al., 2023). Zhang et al. found that atorvastatin was also able to regulate gut microbiota and promote butyrate production. These protected the function of intestinal barrier and reduced the release of endotoxin as well as harmful bacteria into the circulation, which inhibited neuroinflammation mediated by microglia around the infarct area (Zhang P. et al., 2021). Studies also illustrated that direct exogenous supplementation of probiotics or prebiotics could increase the growth of beneficial bacteria and inhibit the growth of harmful bacteria, which maintained normal intestinal barrier function and increased the levels of neuroprotective metabolites to mitigate the inflammatory response in the brain and gut (Yuan et al., 2021; Savigamin et al., 2022). Experiments about traditional Chinese medicine demonstrated that some traditional Chinese medicine prescription, such as Tong-Giao-Huo-Xue Decoction and Dihuang Yinzi, contained a variety of pharmacological components, which reduced neuroinflammation through regulating intestinal flora to promote the recovery of intestinal immune homeostasis (Zhang et al., 2020; Wang et al., 2022). Kurita et al. illustrated that oral administration of nonabsorbable antibiotics, such as polymyxin B, modulated gut microbiota and improved the outcome of endotoxemia and neuroinflammation in mice with regional cerebral ischemia (Kurita et al., 2020).

Wang et al. found that lipopolysaccharide preconditioning mice had milder splenic atrophy after MCAO, with decreased levels of pro-inflammatory immune cells and increased levels of regulatory B cells in the spleen, which mitigated neuroinflammation and improved neurological function. Lipopolysaccharide preconditioning might activate the anti-inflammatory protective mechanism of the spleen after ischemic stroke (Wang et al., 2018). Intravenous administration of stem cells is also a novel approach to modulate post-stroke inflammation. The spleen plays an important role on stem cell therapy for cerebral ischemia (Wang et al., 2019). Acosta et al. demonstrated that in rats with cerebral ischemia, intravenous human bone marrow stromal cells (hBMSCs) rapidly migrated to the spleen and reduced the levels of TNF-α in the spleen, as well as reduced infarct volume and inflammatory response in infarct area (Acosta et al., 2015). Intravenous administration of human umbilical cord blood cells (hUCBs) could inhibit splenic atrophy, regulate the number and function of T cells in the spleen and reduce the levels of inflammatory cytokines TNF-α and IFN-γ (Wang et al., 2019). An animal experiment illustrated that anti-CD147 antibody could inhibit the inflammatory response of monocyte/macrophage in the spleen after cerebral ischemia in mice and reduce the levels of TNF-α, IL-6 and IL-1β in the spleen (Jin et al., 2019). Another animal experiment showed that systemic administration of anti-TNF agents (XPro1595 and etanercept) could inhibit neuroinflammation by regulating the number of peripheral splenic T cells and reducing the infiltration of granulocytes into the brain (Clausen et al., 2014). Modulating cytokines can regulate peripheral immune responses to exert neuroprotective effects. Wang et al. found that intravenous administration of albumin in MCAO mice reduced the expression of TLR4 in the spleen and increased the levels of regulatory T cells and anti-inflammatory cytokine IL-10 in the spleen (Wang et al., 2013). Chang et al. showed that cocaine and amphetamine regulated transcript (CART) regulated CD4^+^/CD8^+^ ratio in the blood and spleen, reduced the expression of pro-inflammatory factors, and promoted the expression of anti-inflammatory factors in MCAO mice, which reduced infarct volume and improved neurological function (Chang et al., 2011). Targeting the cholinergic anti-inflammatory pathway (CHAIP) for immunomodulation is also a potential method to treat post-stroke inflammation (Duris et al., 2017). Acetylcholine and nicotine are agonists of α7 nicotinic ACh receptors (α7nAChRs). These α7 agonists may stimulate α7nAChRs on macrophages in the spleen and gut to inhibit the synthesis and release of pro-inflammatory cytokines (Hoover, 2017).

## 5 Conclusion

Stroke can lead to the activation of resident immune cells in the brain and peripheral immune cells, and activate inflammatory cascades through a series of inflammatory signaling pathways. Both neuroinflammation and peripheral inflammation play important roles in the pathophysiology of stroke. The interaction between brain and peripheral organs (such as gut, heart, lung and spleen) involves the release of cytokines and the activation of signaling pathways, which further coordinate the mutual communication of central and peripheral immune cells. This can promote or inhibit the inflammatory response after stroke. Therefore, a further exploration of the brain-peripheral crosstalk mechanism will help people to better understand stroke. At the same time, using anti-inflammatory therapy may bring more benefits to stroke patients through intervening brain-peripheral axis to regulating central and peripheral inflammation. In addition, we also guess that the drainage and transportation of the MLVs may play a crucial role in the spread of inflammation after stroke, but more studies are needed to demonstrate the pathophysiological mechanism of central lymphoid tissues in inflammation after stroke.

## Author contributions

LX: Writing – original draft, Supervision. MH: Writing – original draft. CY: Supervision, Writing – review & editing. HC: Writing – review & editing, Supervision.
